# Design aspects of vaginal applicators that influence acceptance among target users

**DOI:** 10.1038/s41598-021-89284-3

**Published:** 2021-05-07

**Authors:** Alyssa J. Bakke, Toral Zaveri, Molly J. Higgins, Gregory R. Ziegler, John E. Hayes

**Affiliations:** 1 Sensory Evaluation Center, College of Agricultural Sciences, The Pennsylvania State University, University Park, PA 16802 USA; 2 Department of Food Science, College of Agricultural Sciences, The Pennsylvania State University, 220 Food Science Building, University Park, PA 16802 USA

**Keywords:** Drug delivery, Human behaviour

## Abstract

Although sensory-guided product design is most traditionally used by food and beverage companies, the approach has widespread application for many other products, including pharmaceuticals and medical devices. Previously, our team used sensory methods to explore preclinical optimization of soft-gel vaginal microbicides. Past clinical trials suggest vaginal microbicides may be an effective means for women to protect themselves from HIV and other sexually transmitted infections, but these microbicides will not work if they are not used due to poor acceptability. Our prior work suggests properties like firmness, size, and shape all influence women’s *willingness to try* soft-gel vaginal suppositories*.* As product insertion is part of the overall experience of using vaginal microbicides, understanding the features of vaginal applicators that appeal to women, and incorporating these insights into vaginal drug delivery systems, may also improve user adherence. Despite widespread use of vaginal applicators, there is minimal public data on women’s perceptions of and preferences for physical applicator features. Other work suggests women want vaginal applicators that are single use, pre-filled, made of plastic, and easy to use, store, and discard. Applicator attributes that may be important to women, such as length, color, or visual appeal, have not been investigated previously. The objective of this research was to understand what physical applicator attributes are appealing to women. Here, 18 commercially available applicators were evaluated by a convenience sample of women (n = 102) for overall liking and perceptions of various attributes (perceived length and width, ease-of-grip, expected ease-of-use, expected comfort inside the body, visual appeal, color liking, and environmental friendliness). Preference mapping using both liking data and attribute data showed attributes such as color, visual appeal, ease of grip, expected ease of use, and expected comfort inside the body drove higher liking ratings for applicators, while perceived length negatively affected liking. In general, plastic tampon applicators contained more positive features and were better liked relative to a cardboard tampon applicator or applicators for insertion of medicated gels or suppositories. Incorporating more desirable features into applicators meant for insertion of vaginal microbicides or other vaginal medications may improve the user experience, and possibly user adherence.

## Introduction

Vaginal microbicides hold great potential to empower women with the ability to prevent sexual transmission of HIV and other STIs, but only if they are actually used by women. Past trials with vaginal microbicide candidates have shown mixed results, and some of the differences in efficacy have been attributed to differential product usage and compliance by women in these studies^1–3^. Real-world effectiveness appears to be strongly influenced by user acceptability. To date, large differences in effectiveness have been observed depending on participant usage and compliance^1–3^. Many factors affect women’s willingness to use vaginal microbicides and adhere to the usage protocols. While we cannot overstate the importance cultural and social factors play in adherence (see^3,4^), product features also play a role^5–7^. Using an iterative process involving qualitative focus groups, quantitative consumer tests, and internet-based conjoint surveys, we have studied how product features affect acceptability and willingness to use suppository prototypes with various sensory properties that could be used to deliver active pharmaceutical ingredients into the vagina^8–16^. It is important to note that this work was all preclinical in nature. In each of the previous studies, women evaluated images, concepts, and/or physical prototypes that they were allowed to hold and touch, but the prototypes have not yet been evaluated inside the body. All suppository formulations prepared in our team were viscoelastic solids with a self-supporting shape/form that would facilitate manual insertion but would also permit insertion with an applicator. Previously, we studied ideal product firmness for insertion both with and without an applicator^9^. Focus groups with women in the United States suggested over half of participants would prefer to insert the product with an applicator^16^. Considering a strong desire for an applicator by some women, we were interested in exploring acceptability of different applicator designs that could be modified to use with the semi-soft suppositories developed in our previous work. Consumer products, including medical devices, need to be tested holistically whenever possible to avoid missing key aspects of the overall use experience (e.g.^17^). For microbicide suppositories, the applicator is part of the overall drug delivery system: that is, a well-liked applicator may increase the likelihood of use, and more critically, a strongly disliked applicator may discourage use.

A wide variety of vaginal applicators are employed for insertion of tampons^18^, medicated suppositories or pessaries, or lubricating gels^19–21^. Prior research has shown that women value vaginal product applicators that are disposable, single use, pre-filled, made from materials like plastic, and are easy to use, store, and discard^6,19,20,22,23^. Women disliked reusable applicators that need to be washed between uses, as they raised hygiene concerns especially in the context of products designed for disease prevention (e.g., microbicides for HIV prevention)^6,19^. Ease of use was also an important factor, especially in markets where vaginal product use is not prevalent^23^ or touching of genitals is less acceptable^24^. Ease of storage was a concern for women with children, who feared children would play with the applicator or might ask uncomfortable questions if they found it^7,23^. Sociodemographic factors across markets also influence trade-offs between applicator features such as price and disposability^22^. For example, design was more important than price for women in the Dominican Republic, whereas the two attributes were equally important for women in South Africa, which those authors attributed to the substantially lower median income for women in South Africa^22^. There are important gaps in our understanding of what women desire from vaginal applicators. While product manufacturers presumably must have some of this data in-house, we are unaware of any publicly available data on how attributes like length, color, material and visual appeal affect applicator acceptability.

To help understand which applicator features were important to women, we used preference mapping—a standard consumer science technique—to assess desirable features of commercially available vaginal product applicators. Preference mapping is a multivariate statistical technique that is widely used to aid the product development process by facilitating a deeper understanding of sensory attributes that influence hedonic or affective responses to products^25–27^. External preference mapping creates a multidimensional representation of both hedonic (affective) ratings and non-hedonic data for the products^28^. Such non-hedonic data may be attribute intensities or magnitudes obtained using trained descriptive panels^29,30^, similarity or dissimilarity measurements obtained using rapid methods such as Check All that Apply (CATA)^31,32^ or sorting^33^, or physical or chemical properties of the products derived from instrumental measurements^34,35^. These non-affective data are then linked to hedonic ratings of the products to elucidate drivers of consumer acceptability.

There has been increasing interest in utilizing sensory data collected from consumers vis-à-vis trained panels^36–38^. For example, sensory (intensity) data obtained from consumers via projective mapping has been compared to more traditional external preference mapping using consumer liking ratings combined with sensory (intensity) data from trained descriptive analysis panels^39^. However, few studies have explored preference mapping using sensory (intensity) data from untrained consumers. This may be due to greater noise in consumer data in the absence of training. In our previous optimization efforts with microbicide prototypes, we have routinely obtained ratings for product attributes such as *perceived effectiveness* and *imagined ease of insertion* from untrained consumers. We have observed good consensus and reproducibility in the scale ratings for measured sensory attributes for different product formulations prepared in our laboratory^17^. Accordingly, in the present study, we have used both hedonic and sensory (intensity) data collected from untrained consumers for external preference mapping of vaginal product applicators. Elucidation of sensory attributes that drive the hedonic response for vaginal product applicators will aid in the development of applicators for insertion of semisoft suppositories or other vaginal products with the goal of improving the user experience.

## Materials and methods

### Participant recruitment

Women (n = 102) were recruited as described elsewhere^9^ to evaluate commercially available vaginal product applicators at the Sensory Evaluation Center at Penn State. All participants were recruited from an existing database that consists of a large number (1200 +) of age diverse men and women local to the State College, Pennsylvania area who had previously expressed an interest in routine testing of consumer products in our facility. All participants met the following criteria for inclusion: a) self-identified as female; b) between 18 and 55 years of age; c) reported having had vaginal sex with a man within the last 12 months; d) were willing to manipulate prototypes with their hands and evaluate them using a computer-guided assessment in an isolated test booth.

### Samples and evaluation

**Figure 1:**
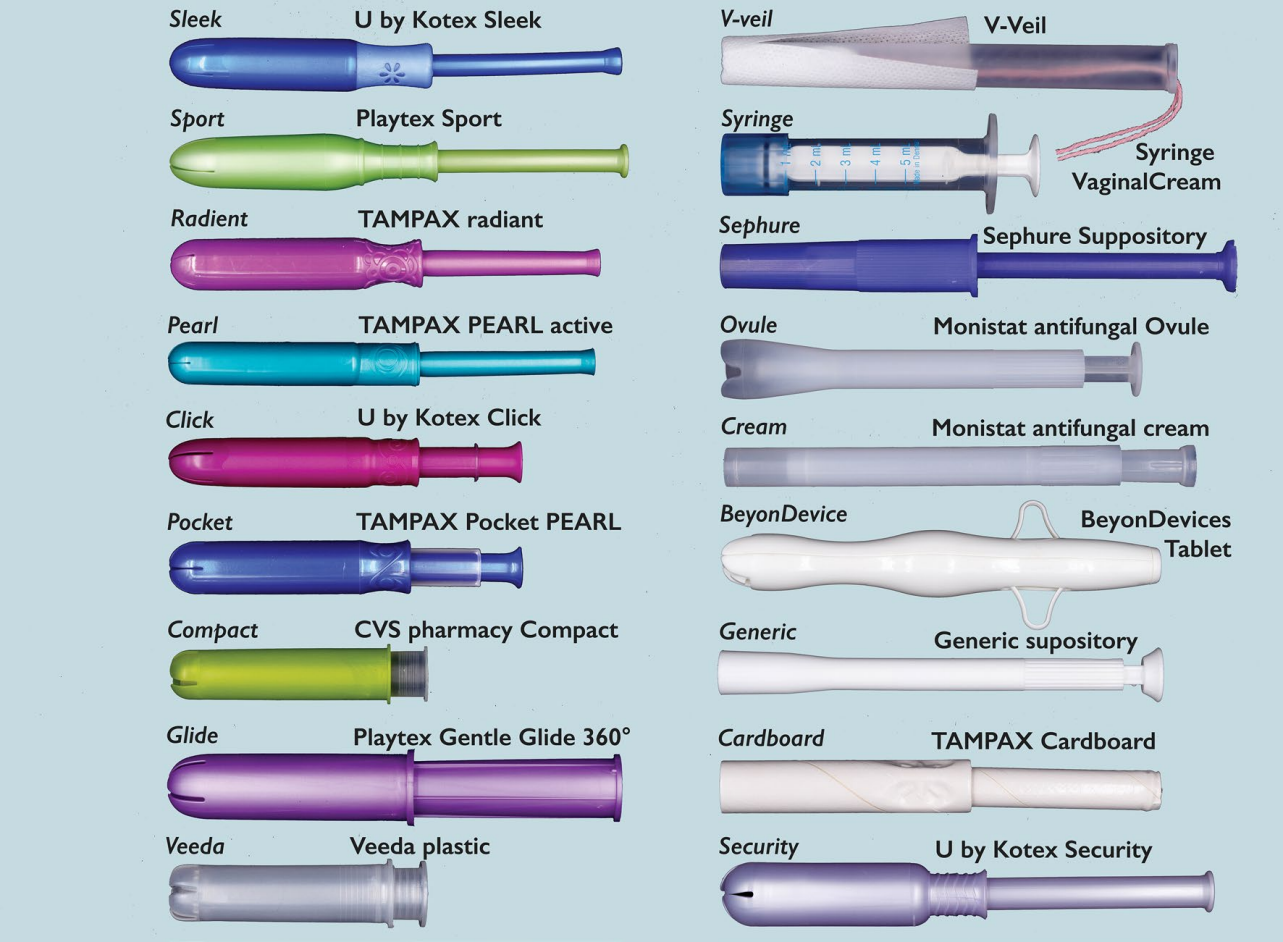
Commercially available applicators used in the study, with abbreviations and photographs.

Eighteen commercially available vaginal product applicators (Fig. 1) were evaluated ex vivo (i.e., women observed and touched the applicators with their hands but did not use them for insertion in the body). Samples included ten plastic tampon applicators, one cardboard tampon applicator, two yeast infection medication applicators, and five vaginal suppository applicators available for purchase without medicine or tampons. The sample set was chosen to provide the largest diversity in applicator features including color, length, width, and material. Since the sample set included applicators used for insertion of both tampons and vaginal gels or suppositories, it was necessary to set a unified context for rating the applicators. Participants were told that we were collecting feedback on the design of applicators that would be used for inserting vaginal suppositories. Images of two of our previously tested suppositories were shown to participants to provide further context and to demonstrate what the suppository might look like (Supplemental Fig. 1). Participants were told that the applicators would eventually be sized to fit the suppositories, so they should not evaluate the applicators for being a good fit for the size / shape of the suppositories depicted. The nature of what the suppositories would be used for (e.g., medication) was not specified. To evaluate the applicator, participants were instructed to pick up the applicator, hold it between her fingers, and imagine using it to insert a vaginal suppository into her body.

In the first round of evaluation, participants rated their overall liking for each applicator using a standard nine-point hedonic scale^40,41^ anchored at 1 = Dislike Extremely on the left end to 9 = Like Extremely on the right end. For ease of presentation, samples were placed in plastic boxes with individual compartments or on trays. In order to evaluate a single sample at a time, samples were covered with blinding code labels. Participants were instructed to check that the blinding code label matched the sample number on the screen, lift the label, evaluate the applicator, and then replace the label in the respective compartment before moving onto the next applicator. Presentation order was counterbalanced across participants using a Williams Design^42^ to control for position and order effects. After women had rated all samples for overall liking, they returned the samples prior to evaluating the applicators again for their sensory properties. This two-stage evaluation procedure (e.g.^43,44^) was used to minimize cuing the participants to specific attributes that might influence liking for subsequent applicators.

After the first round of evaluation, applicators in the boxes/trays were inspected by research staff to check that the order of samples was correct, and to adjust length of collapsible applicators back to their original packaged length. Boxes/trays were then returned to participants for a second round of evaluation where participants were instructed to lift the label as before, visually inspect the sample without picking it up, and rate the perceived length and perceived width of the applicator using a 100-point visual analog scale. These ratings were made without having participants pick up samples to ensure that plungers were not accidentally depressed when picking up the applicator, as this would affect the perceived length. Next, women were then instructed to pick up the applicator and imagine using the applicator to insert a vaginal suppository and then to rate the applicators for her perceptions of how easy it was to grip, how comfortable she expected the applicator would feel inside her body, how easy she expected it would be to use, visual appeal, and the extent to which she thought the applicator was environmentally friendly. All these ratings were made on 100-point visual analog scales. Verbal end anchors (e.g., not at all comfortable on the left to very comfortable on the right) were provided on the scales, and these were indented at 10% and 90% of the scale to minimize end use avoidance bias. Color liking for the applicators was collected using a 9-point hedonic scale. Table 1 presents the shortened keywords that will be used to refer to each of these attributes in the remainder of the manuscript. Women were also provided open-ended text boxes to share what they particularly liked or disliked about each applicator or to provide any additional comments. Demographics, such as age, race, ethnicity, education level, marital status, prior STI diagnosis, prior unplanned pregnancy, number of sexual partners in past year, and number of vaginal deliveries were collected after panelists completed all evaluations (Supplementary Table 1). Prior experience with other vaginal products was collected using a check-all-that-apply (CATA) question (see Supplementary Table 2). Also, preference related to vaginal applicators, such as preference for type of tampon applicators, preference for applicator/manual insertion for vaginal medication, and preference for reusable applicator were collected (see Supplementary Table 3).

**Table 1:** Measured sensory attributes and keywords used to refer to them in figures and text.

Full sensory attribute	Short keyword
Ease of grip	Grip
Expected comfort in the body	Comfort
Expected ease of use	Ease
Visual appeal	Visual
Color Liking	Color
Environmentally friendly	Environment
Perceived width	Width
Perceived length	Length

### Physical measurement of applicator samples

The length and width of applicators (in cm and mm, respectively) were measured using Vernier calipers to compare the physical measurements with ratings of perceived length and width. For length, two different measurements were recorded: the first measurement was the packaged length (i.e., the length of the product upon opening the package) while the second measure was the length of the barrel (the portion of the applicator that would contact the vaginal canal during insertion). For width, two different measurements were recorded: width at the insertion end (vaginal contacting surface) and width at the point on applicator where it would be gripped (grip surface) were measured.

### Statistical analyses

Data were collected using Compusense Cloud (Guelph, Canada) and analyzed using Compusense and R studio version 0.99.491^45^. Packages SensoMineR^46^, and FactoMineR^47^ within R studio were used. Differences in liking and other sensory attributes between the applicators were tested using mixed model ANOVA, with sensory rating as the dependent variable, participant as a random effect, and applicator type as a fixed effect. Fisher’s Least Significant Difference (LSD) was used for post hoc comparisons between applicators with *p* < 0.05 considered significant. An external preference map was generated using Principal Component Analysis (PCA) on the individual liking ratings and mean ratings of the other sensory attributes to determine which sensory attributes drove applicator liking.

### Ethics statement

Participants provided informed consent prior to participation. All study procedures complied with all relevant ethical and legal guidelines, including the Declaration of Helsinki and US federal law. The study protocol was approved by the Institutional Review Board at the Pennsylvania State University. Participants received a small cash incentive for their time.

## Results and discussion

**Figure 2:**
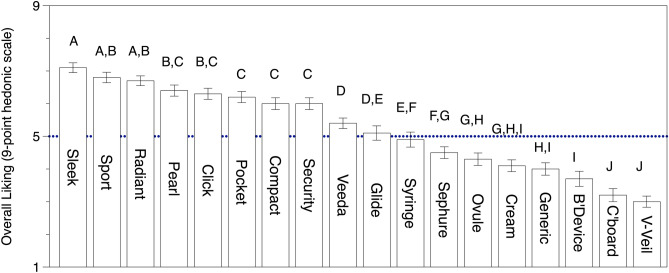
Overall liking means and standard errors (bars) for all 18 applicators measured using a classical 9-point hedonic scale, where 1 = Dislike extremely and 9 = Like extremely, and 5 is a neutral point (‘neither like nor dislike’), ordered with higher liked applicators to the left of the plot. Mean ratings with an upper case letter in common are not significantly different in liking at α = 0.05 (Fisher’s LSD). Most of the products to the left are plastic tampon applicators while those to the right are plastic medication applicators; a cardboard tampon applicator also falls on the right.

**Figure 3:**
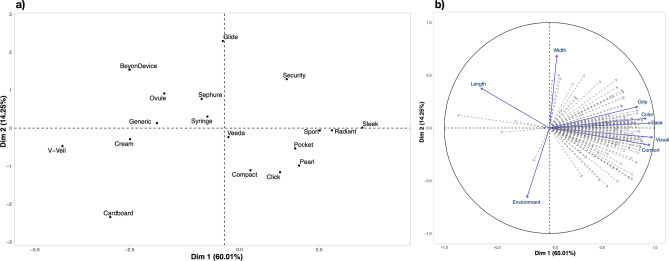
PCA biplot for the external preference map obtained from liking ratings and attribute ratings. Panel (**a**) shows the product plot using the first two principal components; that is, it shows the similarity of each product in relation to the others when all sensory attributes are reduced into two dimensions in PCA. Panel (**b**) shows the attribute and consumer liking loadings; that is, each participant’s liking vector is shown as a gray dashed line, while each sensory attribute vector is shown as a solid blue line, using the same two PC axes (i.e., the first two dimensions from the PCA, which explained 74.26% of the variance). Comparison of the consumer liking vectors to the sensory attribute vectors reveals attributes that drove liking/disliking. For example, products on the right side of the plot in Panel (**a**) were most liked (as shown by the dashed gray vectors in Panel (**b**) for each participant), and these products were in the opposite direction of the perceived length vector in Panel (**b**). Greater liking for products on the right side of Panel (**a**) is wholly consistent with means in Fig. 2. Collectively, the preference map indicates greater overall liking associates with scores for grip, color, ease, visual liking, and expected liking in the body, implying the increase in product liking was driven by specific product features and not merely familiarity. Also, the cardboard tampon applicator was perceived as being more environmentally friendly, but this was insufficient to drive an increase in liking for this disliked product.

**Figure 4:**
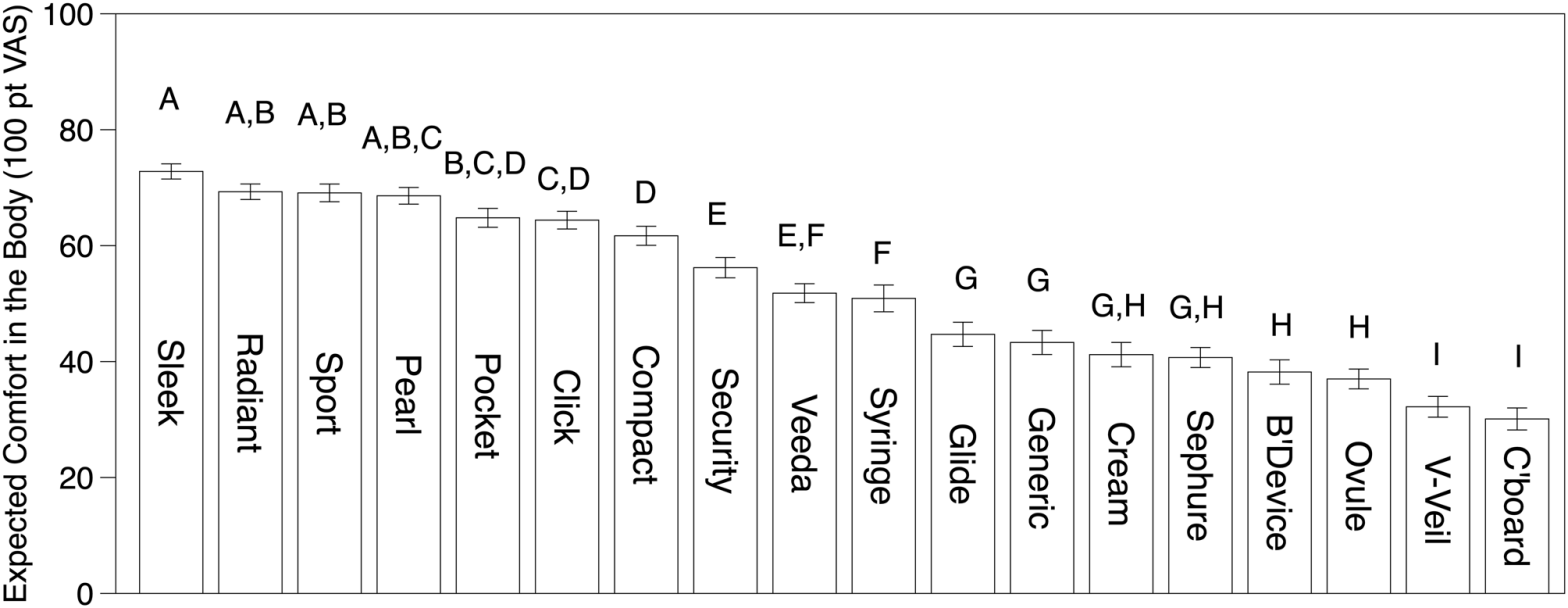
Means and standard errors for expected comfort inside the body. Short applicator names correspond to Fig. 1. Ratings were collected on a 100-point visual analog scale where verbal anchors were placed at 10 (Not at all comfortable) and 90 (Very comfortable); a midpoint was not provided. Mean ratings with a letter in common are not significantly different at α = 0.05 (Fisher’s LSD).

In general, plastic tampon applicators were better liked than the other applicators (Fig. 2). Most of the plastic tampon applicators had an overall liking score of 6 or higher (on a scale from 1 to 9, with neutral at 5), while the applicators for vaginal cream/gels were disliked, as they had overall liking scores below 5. The higher scores generally observed for the plastic tampon applicators could conceivably represent a familiarity effect. That is, ~ 80% of the women in the study had used tampons while far fewer had used vaginal medications, so they may simply be more familiar with tampon applicators than with medication applicators. However, we argue this result may also reflect design features common among the plastic tampon applicators. It is unsurprising tampon manufacturers would be invested in understanding women’s applicator preferences, since tampons are intended for monthly usage while in general medications would be used more sporadically. As anticipated, external preference mapping provided novel insights into the sensory attributes that are important to women’s acceptability of vaginal applicators. The principal components analysis (PCA) biplot for this analysis is shown in Fig. 4. Panel A shows the similarity of each product in relation to the others when all sensory attributes are reduced into two dimensions via PCA. Panel (**b**) of the biplot shows each participant’s liking vector as a dashed line and the vector of each sensory attribute is represented as solid line. Critically, the two halves of the biplot are shown on the same two dimensions from the PCA, so comparing the placement of the liking vectors to the placement of the vectors representing sensory attributes illustrates which sensory attributes drove liking for the applicators. In doing so, we can see the external preference map PCA biplot largely confirms the findings from the ANOVA model shown in Fig. 3—that is, plastic tampon applicators were more liked while the applicators used for delivery of vaginal medications were less liked by consumers. However, the biplot also provides additional insight into *why* these applicators were well liked. Specifically, applicators that were well liked were expected to be comfortable in the body and easy to use and grip. High visual appeal and a better liked color were also desirable features in an applicator; conversely, perceived length was negatively associated with liking scores (i.e., the length vector is in the opposite direction as the better liked products in the biplot). Collectively, this suggests that greater liking is not merely a function of familiarity, but rather evidence that common design features, not familiarity, are driving women’s preferences. These product features are discussed in greater detail below.

### Expected comfort in the body

The applicators that scored highest for expected comfort in the body were all rounded at the insertion end and had a smooth surface throughout (see Fig. 1 for product images and Fig. 4 for mean ratings for expected comfort in the body). Notably, plastic tampon applicators were much more likely to have these features, with higher scores compared to other applicators. On the other hand, applicators like Ovule, Cream, Generic, BeyonDevice, and the Cardboard tampon applicator that were blunt at the insertion end were rated lower for expected comfort in the body. The cardboard applicator scored the lowest for expected comfort in the body; indeed, many women commented that the combination of the material and lack of a smooth rounded end would be uncomfortable or even painful during insertion. One representative statement from the open text comments was ‘I hate cardboard applicators. I think they are very uncomfortable and hurt to use.’ Further, this statement suggests that prior experience with particular applicators (negative in this case) are important contributions to women’s ratings. Also, comments indicated women were unsure if a cardboard applicator could stand up to moisture from the suppositories. Notably, participants also differentiated between rounded-tip plastic applicators depending on whether the petal-like tips were open or closed prior to insertion. They preferred the closed tips, as they feared an open petal may catch or be uncomfortable during insertion. For example, in reference to the Compact applicator, one woman stated ‘I do not like the tip, I feel it would "catch" the inside skin or pinch it.’ The Ovule applicator was rated especially poorly for expected comfort in the body. This applicator was not only wide at the insertion end, but also had an expanded ‘flower-like’ contacting surface meant to hold a suppository. Yet again, text comments indicated some women feared plastic edges would be uncomfortable upon insertion. It is worth noting that this particular applicator is sold with a tapered, smooth suppository that comes in a separate, individual package. It is possible that once the ovule is loaded into the applicator, women would perceive it has having a rounded end. Preloading applicators may help address this concern and have other additional benefits. In prior focus groups, women expressed concerns with manipulating the ovules while loading the applicator^16^. Statements about the BeyonDevice deserve specific comment. Women feared this product may catch in the body and be painful. Many women used words like intimidating, scary, and fearful in reference to it, with one woman even saying it looked like a ‘torture device’ and another calling it a ‘monster’. This applicator was purchased online, and was probably unfamiliar to our participants; presumably, such unfamiliarity may have also played a role in women’s perceptions of this product.

### Ease of grip and use

**Figure 5:**
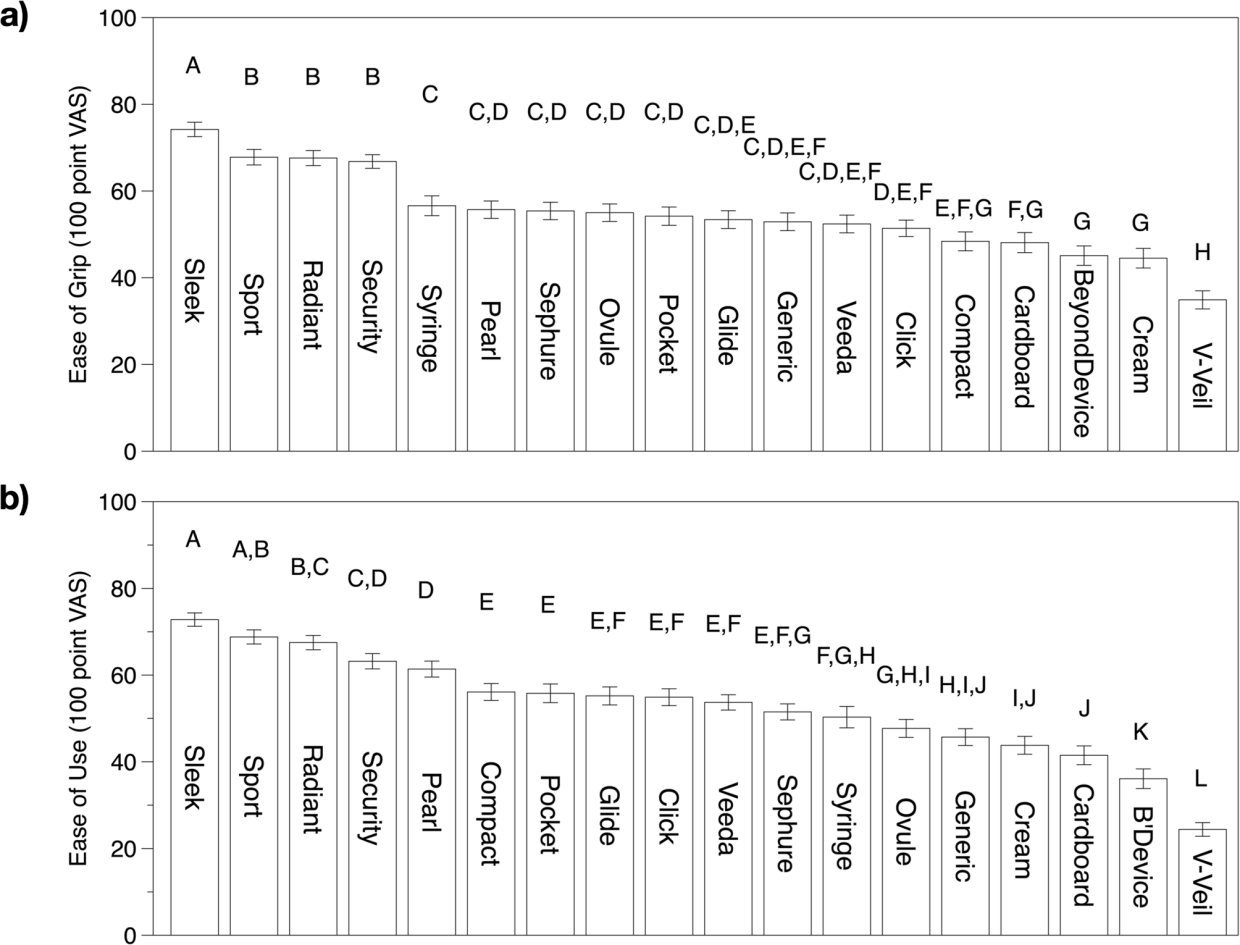
Means and standard errors for *ease-of-grip* ratings (**a**) and *ease-of-use* ratings (**b**). Short applicator names correspond to Fig. 1. Ratings were collected on a 100-point visual analog scale where verbal anchors were placed at 10 (Not at all easy) and 90 (Very easy); a midpoint was not provided. Within a panel, mean ratings with a letter in common are not significantly different at α = 0.05 (Fisher’s LSD).

Applicators with textured grip surfaces that were thinner than the barrel tended to score highest for both ease-of-grip and ease-of-use (see Fig. 5). More than half the participants specifically commented that they liked the rubberized grip of the Sleek applicator, and over 20% of the women commented that they liked the textured grips of Sport, Radiant, and Glide applicators, consistent with the quantitative ease of grip ratings. The V-Veil was one of the least liked applicators: based on text comments from participants, a major reason was the lack of a gripping surface, along with confusion regarding how the applicator worked. While a textured grip surface was generally positive, there was one notable exception. Participants did not appear to like the ridges on the Sephure applicator with one woman writing, ‘I do not like the open end nor do I like the ridges at the top. I do not think the ridges would feel comfortable.’ Notably, there is key difference between the Sephure applicator and the other applicators with textured surfaces. The other applicators are designed so the textured grip surface is narrower than the barrel, creating a clear boundary between the gripping surface and the insertion surface. Thus, the textured surface would not be felt inside the body, whereas the textured surface on the Sephure applicator is integrated into the barrel portion of the applicator that would be inserted into the body.

### Color liking and visual appeal

**Figure 6:**
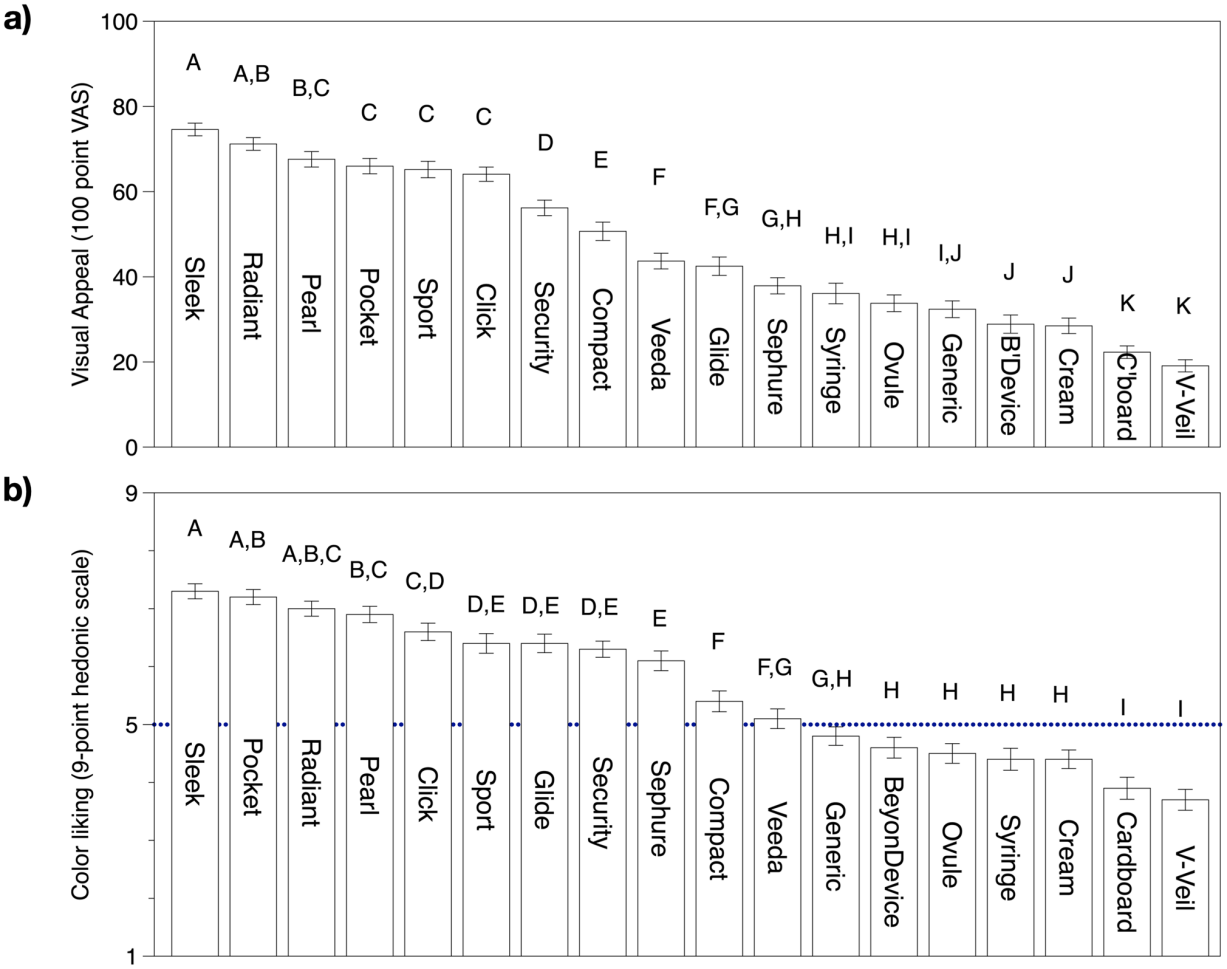
Means and standard errors for (**a**) visual appeal (VAS rating) and (**b**) color liking (9 pt hedonic scale). Short applicator names correspond to Fig. 1. Ratings for visual appeal were collected on a 100-point VAS where verbal anchors were placed at 10 (Not at all appealing) and 90 (Very appealing); a midpoint was not provided. Coloring liking was obtained with a 9-point hedonic scale where 1 = Dislike extremely and 9 = Like extremely. Within a panel, mean ratings with a letter in common are not significantly different at α = 0.05 (Fisher’s LSD).

Colorful plastic tampon applicators scored higher than the white/transparent applicators in both visual appeal and color liking (Fig. 6). Generally, dark-colored applicators scored higher in color liking compared to light-colored or white/transparent applicators. That said, women did not like the green colored applicators, and some commented on negative connotations between green color and infections: ‘It is a color I would call infection green’ and ‘The color is a little retro, and I don't want to put lime green/yellow things into my vagina.’ Some women also commented specifically that they did not like the white/translucent color of the applicators. For example, one participant commented in reference to the Monistat cream applicator: ‘The color is translucent and very obvious to tell if dirty, which might gross me out if after use I saw any sort of vaginal residue on it.’ In reference to the Monistat Ovule applicator, another participant commented: ‘It is a bit boring. Just white/opaque. Looks "dated" not very modern looking. Looks very medicinal.’ In reference to the generic suppository applicator, one participant wrote, ‘Too long and thin and the hard plastic makes it seem to medical-like and not appealing.’ Regarding the BeyonDevice, one participant wrote: ‘It feels clinical because of the white color and the strange shape.’ Some women commented they preferred the sheen/pearly finish of some applicators, and this was particularly seen in the case of the Veeda applicator, which was liked in spite of its white color. For example, in reference to the Veeda applicator, one participant wrote: ‘Pertaining to color, I like the shimmer—it improves a basic clear color.’

### Length and width

**Figure 7:**
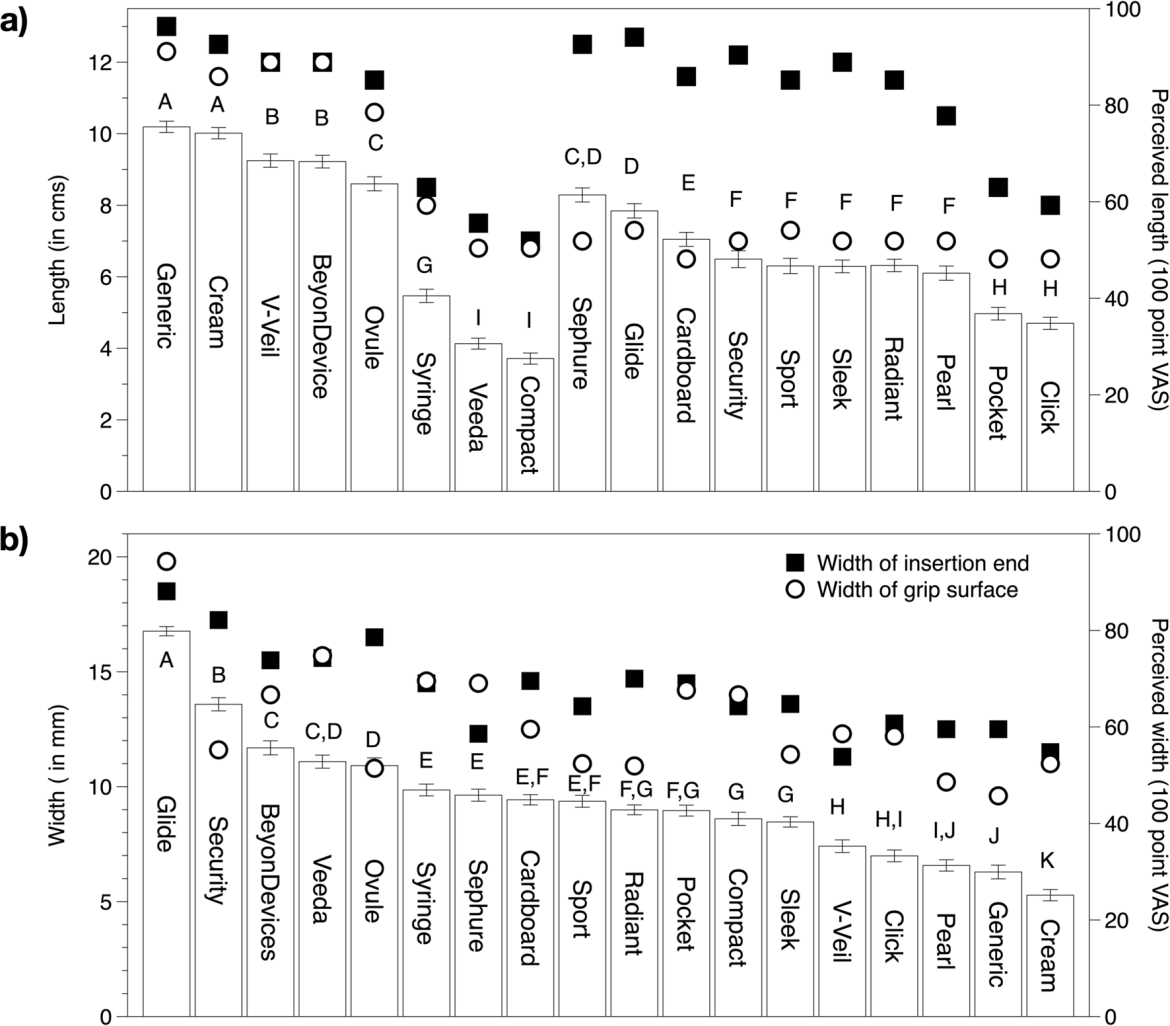
Comparison of physical dimensions with perceived length and width, as measured on 100 point visual analog scales with indented semantic anchors at 10 (Very short/thin) and 90 (Very long/thick). Panel (**a**) shows the influence of physical barrel and packaged length on perceived length. Open bars and error bars indicate perceived length on the VAS for length; physical length (in cm) for each applicator is shown as packaged (solid squares) and barrel length (open circles). Notably, the 8 products on the left had similar lengths for barrel length and package length (overlapping circles and squares), and these generally corresponded to the perceived length (open bars). In contrast, the 10 applicators on the right side had substantial differences between packaged length and barrel length. Panel (**b**) shows how perceived width is influenced by physical dimensions of the insertion end and grip surface. Open bars and error bars indicate perceived width on the VAS; physical width (in cm) are shown for the insertion end (solid squares) and grip surface (open circles). As expected, perceived width was more closely associated with the physical width of the insertion end than the grip surface width. Within in a panel, mean ratings that have a letter in common are not significantly different at α = 0.05 (Fisher’s LSD).

Our data suggest it is not the actual length but rather the perceived length which influences liking; critically, perceived length depends on the applicator design. Specifically, by varying barrel length, plunger diameter, and range of motion of plunger across the length of the barrel, the perceived length can be manipulated. For example, the packaged length of vaginal medication applicators such as the Ovule and Cream were comparable to tampon applicators (Glide, Sport, Sleek and Radiant), yet the perceived length was significantly lower for the tampon applicators (Fig. 7). This may be due to the women focusing only on the barrel of the tampon applicators while evaluating length, as the plunger is significantly smaller in diameter and it collapses inside the barrel upon use. The long plunger of the Sephure applicator was particularly worrisome, as several women were concerned the plunger would over-extend and hurt them. Similarly, and as might be expected, women made ratings of perceived width based on the physical width of the insertion end rather than the width at the grip surface (see Supplemental Fig. 3).

## Conclusion

One recent study looking at market strategies for microbicide introduction suggested a different branding of the product may aid in better product adherence^48^. NIAID Research on Microbicides lists acceptability and desirability as optimal characteristics of microbicides. Taking into account features, including applicator design, that positively affect consumer usage experience may increase the likelihood that women will want to use microbicides. Thus, applicators should be viewed as one part of the entire product experience. Our results indicate applicator features strongly influenced women’s perception of and liking for vaginal applicators. Here, women liked applicators that they expected to be comfortable in the body, easy to use and grip, and with higher visual appeal. These applicators tended to be darker-colored plastic applicators with rounded tips, textured grips, and thin plungers. Applicators provided with commercial vaginal medications or during microbicide gels during clinical trials tend to be white or transparent and look medicinal. While applicator designs to date have understandably focused on safety, ease of use, cost and function (delivery of the medication at the desired site)^20,49,50^, our data suggest features like comfort in the body, ease of use, and aesthetics should also be taken into account. Visual product features of applicators such as shape, color, surface appearance, and grip surface connote symbolic meanings regarding the products and their quality (i.e., “meaning making”;^51^), which may encourage or discourage women from using them.

Such attributes may be especially important for an applicator that has the potential to be used peri-coitally. In focus groups conducted with prototypes of semisoft suppositories, many women desired a product that could be used one-time immediately prior to intercourse while maintaining efficacy, since most of their sexual encounters, particularly the riskiest sexual encounters, were unplanned. It is unclear if such a product is technically feasible, but the consumer desire for one should not be ignored. During this same focus groups, some women suggested these products could be incorporated as part of foreplay, with partners assisting with product insertion^15^. This is also corroborated by an earlier vaginal gel study where women reported that the gel played a part in foreplay^6^. Women might be particularly aware of aesthetics and the associated connotations of applicators if they will be seen by sexual partners, and this also has the potential increased worries about stigma. Stigma associated with HIV/AIDS impedes prevention efforts, including biomedical interventions technologies such as HIV vaccines and microbicides^52–54^. Applicators that bring up associations with medicine or treatment of disease may be especially unwelcome within the context of intercourse and stigma. Making such products more desirable, which may include making them more aesthetically appealing, might help ‘de-medicalize’ them. We believe designing the entire product experience to be more appealing to women will positively impact device efficacy. On the other hand, previous research has shown that women derive meaning about efficacy from design features, as well, so a more medical appearance could enhance perceptions of efficacy. Additional work will be needed to confirm this empirically. Future research could also help discern how sensory and aesthetical factors regarding both products and applicators fit into the larger scheme of willingness to use and adhere to microbicide protocols. While prior work found good agreement between the current participant pool and a higher risk more diverse sample^15^, further work in other populations is needed to confirm present results.

## Supplementary information

Supplementary Information.
