# Supplementary material for: Design aspects of vaginal applicators that influence acceptance among target users
Source: Sci Rep. 2021 May 7;11:9802. doi: 10.1038/s41598-021-89284-3 (PMC8105386; doi:10.1038/s41598-021-89284-3)
Supplement: Supplementary file 1 — Supplementary Information. [file 41598_2021_89284_MOESM1_ESM.pdf]

Supplementary Materials for:

"Design aspects of vaginal applicators that influence acceptance among target users"

Alyssa J. Bakke[1,2], Toral Zaveri[1,2], Molly J. Higgins[1,2], Gregory R. Ziegler[2], and John E. Hayes[1,2,*]

[1]Sensory Evaluation Center and [2]Department of Food Science

College of Agricultural Sciences

The Pennsylvania State University,

University Park, PA 16802

*Corresponding Author:

Dr. John E. Hayes
Department of Food Science
Pennsylvania State University
220 Food Science Building
University Park, PA 16802
814-863-7129 (voice)
jeh40@psu.edu
Twitter: @TasteProf

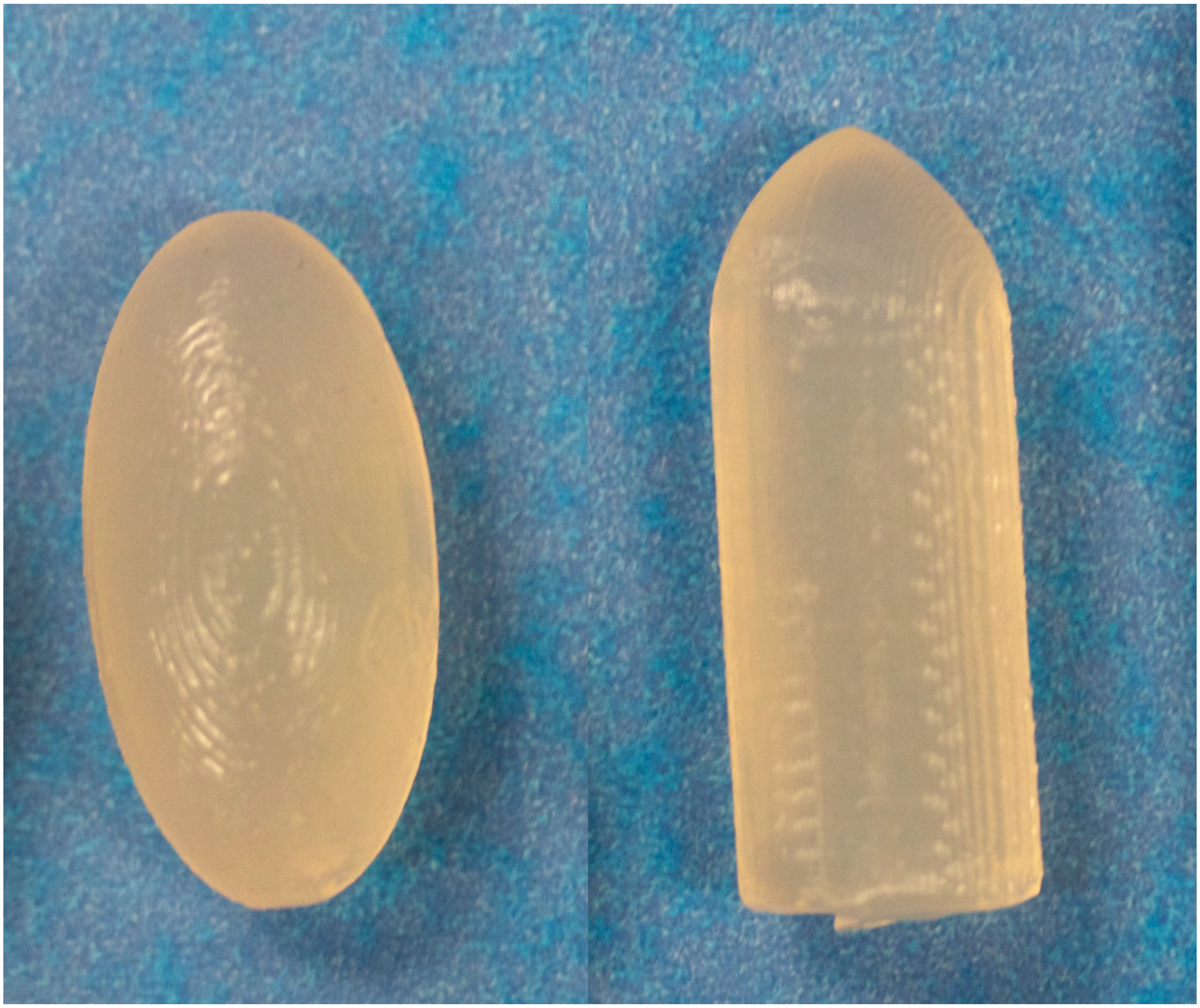

Supplemental Figure 1: Images of semi-soft suppositories presented to study participants as examples of a product that could be inserted with the help of the applicators being evaluated in this study. Participants were told the suppositories could be optimized for individual applicators later and they should not evaluate the applicators in terms of being a good fit for the size / shape of the suppositories shown here.

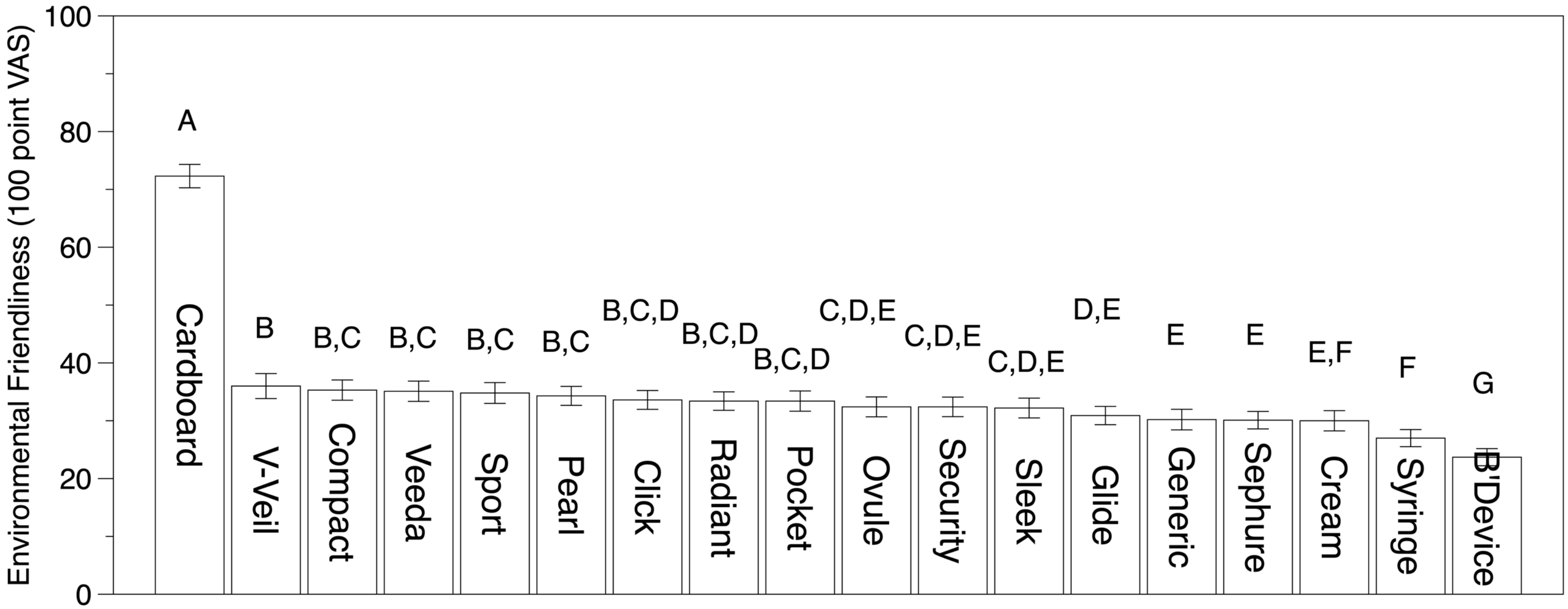

Supplemental Figure 2: Means and standard errors for environmental friendliness on a 100 point VAS where verbal anchors were placed at 10 and 90; a midpoint was not provided. Mean ratings with a letter in common are not significantly different at α=0.05 (Fisher's LSD).

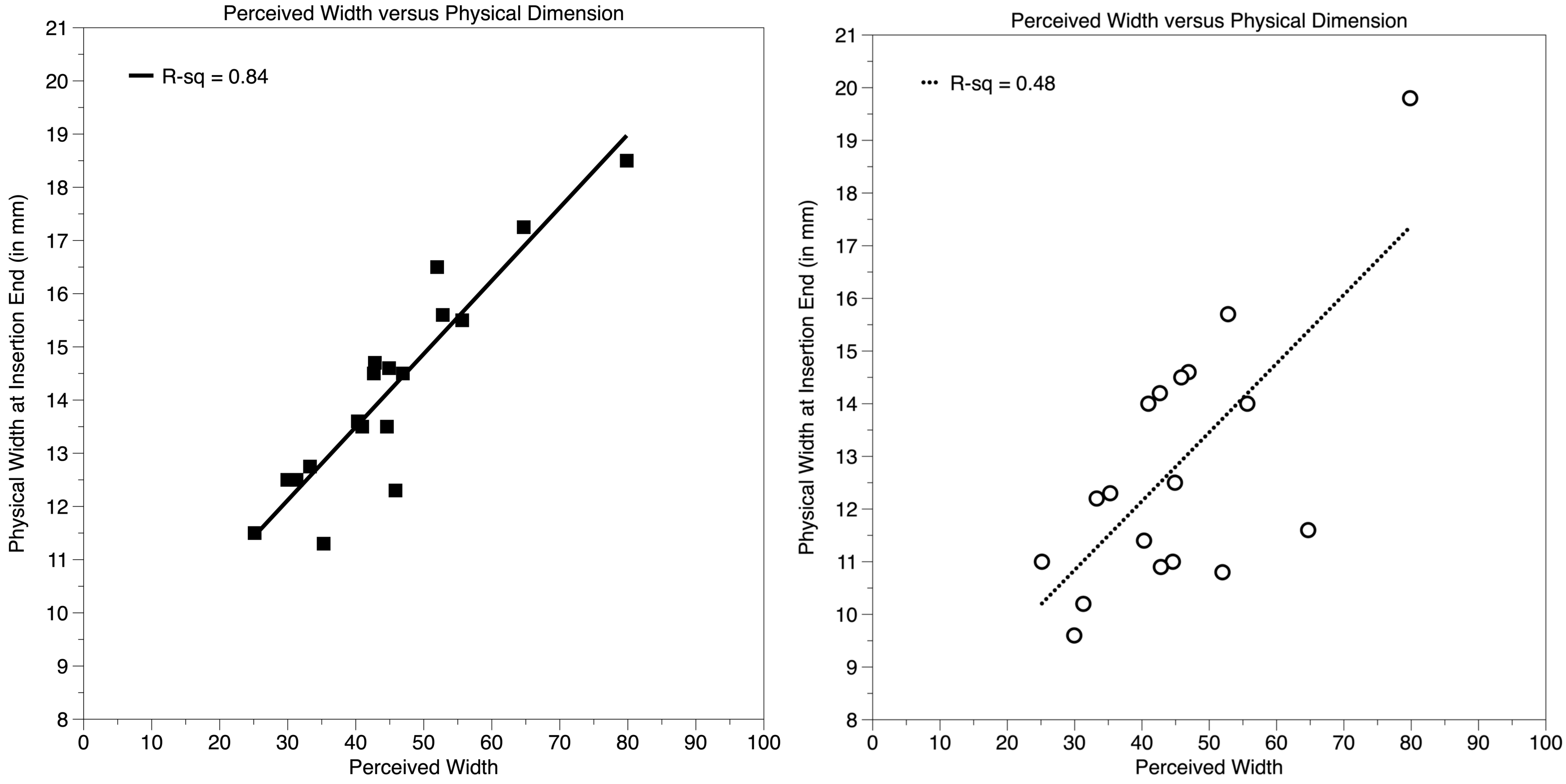

Supplemental Figure 3: Correlation between perceived width (100 point VAS) and the physical dimensions of the insertion end (left) and the grip surface (right).

Supplementary Table 1: Demographics (n=102).

Age	% Participants		Marital Status	% Participants
18-24 years old	31.4		Now married	42.2
25-29 years old	21.6		Divorced/Separated	7.8
30-34 years old	8.8		Never married	50.0
35-39 years old	15.7		Vaginal Births	
40-44 years old	16.7		None	71.6
45-50 years old	5.9		One	10.8
Race			Two	12.7
Caucasian or White	93.1		Three or more	4.9
Black or African American	2.9		Sexual Partners in the past 12 months	
Asian	2.9		One	77.5
More than 1 race	1.1		2 to 3	14.7
Ethnicity			4 to 5	3.9
Not Hispanic or Latino	96.1		6 to 10	2.9
Hispanic or Latino	3.9		More than 10	1.0
Highest Level of Education			STI Diagnosis	
High School/GED	10.8		Yes	9.8
1 or more years of college, no degree	29.4		No	90.2
Associate's degree	13.7		Unplanned Pregnancy	
Bachelor's degree	25.5		Yes	14.7
Master's degree	15.7		No	85.3
Professional degree	2			
Doctorate degree	2.9			

Supplementary Table 2: Prior vaginal product usage from a Check All That Apply (CATA) question, so column totals may exceed 100% (n=102).

Vaginal products used	% Participants
Vaginal contraceptive products such as NuvaRing®	6.9
Spermicidal gels and films	3.9
Yeast infection medicines such as [illegible]	22.5
Douche	4.9
Menstruation products such as tampons	79.4
Lubrication products such as KY® gels, liquibeads [illegible]	32.4

Supplementary Table 3: Vaginal product applicator preferences (n=102).

Do you prefer tampons with	% Participants
Plastic applicator	76.5
Cardboard applicator	2.9
No applicator	3.9
I have no particular preference	6.9
I don't use tampons	9.8
If you were to use a vaginal product for medication delivery would you	
Only use an applicator	38.2
Prefer to use an applicator	53.9
Don't have a preference	4.9
Prefer to use fingers	2.0
Use only fingers	1.0
I would use a reusable applicator to insert vaginal product for medication delivery	
Strongly agree	6.9
Somewhat agree	31.4
Neither agree or disagree	10.8
Somewhat disagree	22.5
Strongly disagree	28.4
